# The New Remedies

**Published:** 1890-05

**Authors:** 


The New Remedies; A Bi-Monthly Epitome of Progress in Homœopathic Materia Medica and Therapeutics. Edited by James E. Gross, M. D. Gross & Delbridge, 48 Madison Street, Chicago. Subscription, 50 cents a year.

The fourth number of this new journal is before us.

It contains sixteen pages. The title-page informs us that the contributors are Edwin M. Hale, M. D., Robert N. Tooker, M. D., J. H. Buffum, M. D., A. C. Cowperthwaite, M. D., J. R. Kippax, M. D., Clifford Mitchell, M. D., N. B. Delamater, M. D., W. M. Stearns, M. D., Henry Sherry, M. D., and C. A. Williams, M. D.

As its name indicates, this journal is devoted to the announcement and latest information upon new medicines. Unfortunately, it seems to recommend these remedies to be used empirically. There are but few pathogenetic indications for any remedy to be found in these pages. We are unable to discover any homœopathic teachings in it at all. Still, a few interesting items of information about drugs may be gleaned. W. M. J.

